# The psychosocial outcomes of older parenthood in early to mid-childhood: a mini-review

**DOI:** 10.1093/humrep/dead070

**Published:** 2023-04-10

**Authors:** Joanna Lysons, Vasanti Jadva

**Affiliations:** Centre for Family Research, University of Cambridge, Cambridge, UK; EGA Institute for Women's Health, University College London, London, UK

**Keywords:** postponement transition, advanced parental age, advanced maternal age, advanced paternal age, psychology, family functioning, child adjustment, medically assisted reproduction

## Abstract

Recent decades have seen a global trend towards delaying parenthood, referred to as the ‘postponement transition’. Whilst there is plentiful research regarding obstetric and paediatric outcomes related to delayed parenthood, relatively little is known about the psychosocial outcomes associated with advanced parental age during early and middle childhood. This mini-review examines the current literature regarding the psychosocial functioning of families headed by older parents. First, we give an overview of the literature that examines the psychological wellbeing of older first-time parents. We then review the literature regarding the quality of the parent–child relationship in older parent families. Finally, we discuss the psychosocial adjustment and cognitive development of children of older parents. We conclude with suggestions for future research avenues.

## Graphical Abstract

**Graphical Abstract:**
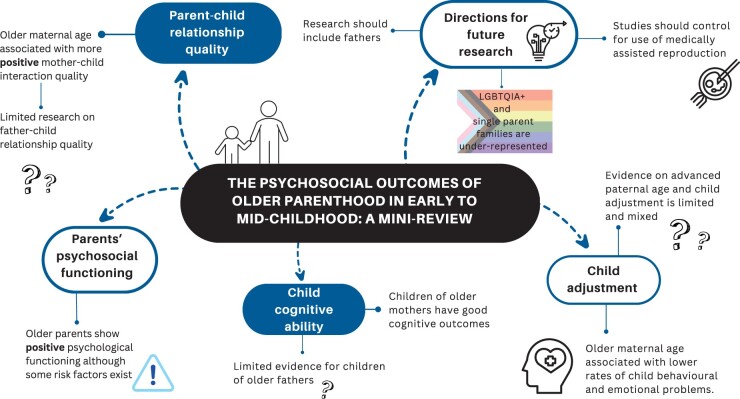
**Whilst older parenthood is associated with both risk and protective factors, the literature demonstrates good psychosocial functioning in older parent families.** Data regarding fathers and single parent or LGBTQIA+ families are very limited.

## Introduction

The average age of first-time parents continues to rise across the globe. In the UK, the average age of first-time mothers rose from 27.7 years in 2010 to 29.1 years in 2020 (ONS, 2021), with a similar pattern observed amongst fathers (ONS, 2019). This trend has been observed in the USA (Matthews and Hamilton, 2009; Khandwala *et al.*, 2017), across continental Europe (Billari *et al.*, 2006; Trillingsgaard and Sommer, 2018; Beaujouan and Toulemon, 2021) and in Asia Pacific (Adachi *et al.*, 2020; Lazzarri, 2021). Within the literature, this move towards delayed parenthood has come to be termed the ‘postponement transition’ (Kohler *et al.*, 2002). Studies cite various reasons for the postponement transition, including changes in economic opportunities and pressures, changing patterns of education and marriage, and the development of reliable, affordable medically assisted reproduction (MAR) (Billari *et al.*, 2006; Khandwala *et al.*, 2017; Beaujouan and Toulemon, 2021).

However, despite this widespread trend towards delayed parenthood, the evidence regarding the developmental, psychological, and socioemotional outcomes of families headed by older parents is patchy. Whilst there exists a reasonably rich literature regarding associations between advanced parental age, particularly advanced maternal age, and obstetric and parent psychopathological outcomes in the perinatal period, less is known about the associations between older parenthood and parent psychological functioning beyond the transition to parenthood (Trillingsgaard and Sommer, 2018; Couture *et al.*, 2021). Similarly, whilst several studies have examined associations between older parenthood and child physical and neuropsychiatric outcomes, fewer have examined aspects of family functioning such as the quality of the parent–child relationship and child socioemotional adjustment. This review aims to summarize the extant literature regarding advanced maternal and advanced paternal age, in relation to family psychosocial functioning.

A review of the literature regarding older parenthood must first acknowledge several pervasive methodological limitations. First, the scholarship does not agree about what constitutes older parenthood, with advanced maternal age operationalized variously as upwards of 30 years (Pollock, 1996; Fergusson and Woodward, 1999), ≥35 (Goisis *et al.*, 2017), to ≥40 (Oldereid *et al.*, 2018). There is even less consensus regarding what constitutes advanced paternal age. Moreover, studies differ in their approach in that some treat parental age as a categorical, rather than a continuous, variable, thereby limiting the ability to detect non-linear associations. When treated as a categorical variable, there is little standardization as to what constitutes young, middle-aged, and older parenthood, although a minority of studies use population means of age at first birth (e.g. Boivin *et al.*, 2009) or theoretical perspectives (e.g. Garrison *et al.*, 1997) to formulate age categories for maternal age. However, this approach is not commonplace, and population means vary from country to country.

Second, advanced parental age is associated with sociodemographic factors known to impact child development and family functioning. For example, contemporary older mothers tend to have a higher education level, be more financially secure, have fewer children, and have been married for longer, compared with their younger counterparts (Mac Dougall *et al.*, 2012; Falster *et al.*, 2018; Imrie *et al.*, 2019). Not all studies control for these potentially confounding variables. A final complication is that samples of older parents will inevitably include couples who have conceived using varying forms of MAR, the experience of which can itself have an impact on parents’ psychological wellbeing (McMahon *et al.*, 1997; Daniluk, 2001). Moreover, some forms of MAR, such as IVF, can result in increased rates of multiple births, which has been found to have implications for parental psychological wellbeing (Vilska *et al.*, 2009; Wenze *et al.*, 2015) and the quality of the parent–child relationship in infancy and early childhood (Glazebrook *et al.*, 2004; Boivin *et al.*, 2005). Some (e.g. Boivin *et al.*, 2009, Jadva *et al.*, 2022), but not all, studies of older parents provide details of, or control for, use of fertility treatment. Consequently, the independent effects of parental age and other demographic factors on family psychosocial functioning can be difficult to disentangle.

## Advanced parental age and parental psychological wellbeing

The few studies investigating the psychological wellbeing of older parents when children are in early to middle childhood yield mixed results. The study by Boivin *et al.* (2009) of first-time parents with school-aged children conceived via various MARs, including IVF, ICSI, and donor insemination, found that older mothers and fathers (≥38 years) expressed significantly less warmth towards, and perceived significantly less warmth from one another, compared to middle-aged and younger parents (<38 years). This finding was replicated by a recent study of 72 families with 5-year-olds conceived via oocyte donation (Jadva *et al.*, 2022), where it was found that parents in the oldest age group (≥45 years at child’s birth) reported poorer levels of couple relationship quality than those in younger age groups (<36, 36–40, and 41–44 years at child’s birth). The authors of both studies note that since expressions of love and affection have been found to decline steadily over the course of a relationship (VanLaningham *et al.*, 2001; Corra, *et al.*, 2009), and since older parents are more likely to have been in their relationships for longer, this finding may be a reflection of this downward trend in affective display, rather than a genuine effect of older parenthood. Boivin *et al.* (2009) also found higher levels of depressive symptoms amongst older mothers and fathers, although the group differences in maternal depression were accounted for by higher depressive symptoms amongst older mothers who had used donor oocytes to conceive. Conversely, in a sample of 3370 women aged 15–43 at first childbirth, Joo *et al.* (2019) found older maternal age at first birth to be associated with lower levels of depression, although it must be noted that no information was available about how old the children were when the data were collected. Similarly, in a sample of 592 first-time mothers, McMahon *et al.* (2015) found older maternal age (≥37 years) to be unrelated to the prevalence or timing of major depressive episodes from the postnatal period up to 2 years post-partum. Jadva *et al.* (2022) found that the parents in the oldest age group reported higher levels of parenting stress than those in younger age groups, but found no differences on measures of parental anxiety or depression, with the majority of parents in all age groups reporting low levels of depression and anxiety.

Thus, quantitative studies of psychopathology in older parents of school-aged children appear to provide somewhat inconclusive results; qualitative studies may yield further insights into parents’ experiences of older parenthood. Some studies report that older mothers perceive a lack of social support throughout the early years (Bornstein *et al.*, 2006; Mori *et al.*, 2014) and into childhood (Meyer, 2020; Jadva *et al.*, 2022). This lack of social support has been found to be compounded by the fact that older parents of young children are also more likely to have elderly parents of their own needing support. Thus, older parents of young children may represent a ‘sandwich generation’ wherein there is pressure to simultaneously provide support to both young children and elderly dependents (Meyer, 2020).

Despite its increasing visibility in society, a significant level of stigma still surrounds older parenthood (Campo-Engelstein *et al.*, 2016). Qualitative studies have demonstrated various sources of such stigma, including concerns from healthcare professionals (Meyer, 2020; Nottingham-Jones *et al.*, 2020), judgement from friends (Friese *et al.*, 2006; Jarvie *et al.*, 2015; Jadva *et al.*, 2022), and negative portrayals of older parents in the wider media (Jarvie *et al.*, 2015; Campo-Engelstein *et al.*, 2016). Some parents have also reported concerns that their children may be singled out by their peers due to having significantly older parents than others (Mac Dougall *et al.*, 2012). It is therefore possible that perceived or actual stigma around older parenthood may impact older parents’ psychological wellbeing, which may in turn be felt across the wider family system. This said, it is important to note that some qualitative studies have demonstrated overtly positive outcomes of parenting at an older age. Parents in Jadva *et al.* (2022)’s study reported several positive aspects of older parenting, such as feeling they had more resources and commitment to dedicate to parenting than they would have had at a younger age. Some parents also reported feeling that being the parent of a 5-year-old kept them feeling young; this is echoed by findings from Mac Dougall *et al.* (2012), where some parents reported that having relationships with younger parents had the benefit of keeping them more culturally relevant.

## Advanced parental age and parent–child relationship quality

Very few studies focus specifically on the quality of the parent–child relationship in relation to parental age, and those studies that do so, focus almost exclusively on the mother–child relationship. Boivin *et al.* (2009) found that older maternal age was significantly associated with a more positive mother–child interaction; however, the contrasts between young, middle and older maternal age categories were non-significant. Similarly, a recent Dutch cohort study found that, amongst 4741 mothers aged 17–48 years at birth, increased maternal age was related to less frequent use of verbal and physical sanctions with their child when the children were aged 7 and 11 (Trillingsgaard and Sommer, 2018). Analysing longitudinal data from a national cohort study, Schlomer and Belsky (2012) found that older maternal age was associated with greater levels of maternal sensitivity when children were aged 10 years, which in turn predicted lower levels of child-perceived mother–child conflict when children were in adolescence. It must be noted that the mothers in Schlomer and Belsky (2012)’s study were not all first-time mothers; however, birth parity was controlled for in all analyses. The limited quantitative evidence therefore suggests a potentially protective effect of advanced maternal age on mother–child relationship quality.

To our knowledge, three qualitative studies have examined representations of parent–child relationship quality in older parent families. Chen and Landau (2015)’s study of 20 first-time mothers (mean age at birth = 45 years) found that mothers felt that their older age negatively affected their parenting style and, subsequently, their relationship with their child. Moreover, those who perceived themselves as more anxious, over-protective and critical in the context of the mother–child relationship attributed these characteristics to being an older first-time parent. These mothers also reported their children as being ‘preoccupied’ with their parents’ mortality as a result of their older age. This finding is echoed in Mac Dougall *et al.* (2012)’s qualitative study of 61 first-time parents aged ≥40 years, where both mothers and fathers worried that they would have less of their total lifespans to spend building relationships with their children. Some parents in this study also reported that they had smaller energy reserves to devote to parenting their young, energetic children. Similarly, a minority of parents in Jadva *et al.* (2022)’s study similarly reported that they questioned their own parenting skills because of their older age, with some reporting that they felt ‘tired and slow’ due to their age.

It is important to note that the qualitative and sometimes retrospective nature of these studies means that no conclusions can be drawn about whether parents’ perceptions of their parenting correspond with their actual parenting practises and the quality of their relationships with their children. However, it is conceivable that greater anxiety around issues associated with older parenthood may impact parenting quality, which may in turn spill over to the parent–child relationship. As noted above, few studies have examined the father–child relationship quality and fathers’ experiences of being an older parent. The literature suggests that men who become fathers at an older age have been found to be more highly involved in parenting than those who become parents at a younger age (Cooney *et al.*, 1993). This is supported by Jadva *et al.* (2022)’s finding that a number of fathers felt that their older age made them more dedicated parents, and that being an older parent meant they had developed better, more well-defined parenting values. Heterosexual older mothers have also been found to share more parenting tasks with their partners when their children are infants (Bornstein *et al.*, 2006). It is therefore possible that interpersonal processes associated with being an older parent may buffer any negative effects on the parent–child relationship of parenting energetic young children at an older age. Together, these findings suggest that advanced parental age is generally associated with good parent–child relationship quality when children are in early childhood.

## Advanced parental age and child adjustment

The literature regarding advanced maternal age and child problem behaviours, known as externalizing problems, points to a consistent protective effect of advanced maternal age. Several cross-sectional (D’Onofrio *et al.*, 2009; Zondervan-Zwijnenburg *et al.*, 2020) and longitudinal (Fergusson and Lynskey, 1993; Saha *et al.*, 2009a; Sutcliffe *et al.*, 2012; Tearne *et al.*, 2015; Trillingsgaard and Sommer, 2018) studies have found an association between increased maternal age at birth and a decreased risk of child externalizing behaviour problems, from the age of 4 through to adolescence. An early cohort study from New Zealand found clear and consistent associations between increased maternal age at birth and a lower risk of mother- and teacher-reported conduct problems when children were aged 8, 10, and 12 (Fergusson and Lynskey, 1993). In order to isolate the unique effects of maternal age, this study controlled for two broad categories of potential confounding variables, one to capture maternal sociodemographic and family history, and the other to capture child sociodemographic and family history since birth. When both categories of covariates were included in the analyses, the size of the association between older motherhood and lower levels of behavioural problems reduced dramatically, yet remained statistically significant.

In their national cohort study of over 2000 Australian mothers, Tearne *et al.* (2015) reported a negative linear relationship between maternal age and offspring behaviour problems, such that increases in maternal age were related to decreases in offspring problem behaviours. This finding has been replicated with large Danish (Trillingsgaard and Sommer, 2018, n = 4741) and British (Sutcliffe *et al.*, 2012, n = 22 504) samples. The findings from the latter study are particularly robust, as the observed association between older maternal age and fewer offspring behavioural problems remained significant after controlling for a wide range of sociodemographic and physiological factors including children’s age and sex, birth weight, birth parity, maternal socioeconomic status and income, paternal age, and family structure. Similarly, Zondervan-Zwijnenburg *et al.* (2020) found that increases in maternal age at birth were associated with a decreased risk of behaviour problems in a sample of over 30 000 Dutch 10- to 12-year-olds. Older maternal age was also associated with lower levels of teacher-reported externalizing problems, although this association weakened after controlling for various sociodemographic variables.

Conversely, Boivin *et al.* (2009) found no group differences in child problem behaviour according to young, middle, or older maternal age. This discrepancy may be attributable to differences in sample size, wherein very small effects may have gone undetected. It is also worth noting that Boivin *et al.* (2009)’s sample comprised only families that were created using MAR. In their study of parents with families created via oocyte donation IVF, Jadva *et al.* (2022) also failed to find any association between older parental age and child internalizing problems. Thus, there is subtle evidence that those who conceive following MAR may have differential outcomes which may not generalize to the broader population. Going forward, it will be important for large cohort studies to acknowledge and pay attention to this factor in subsequent analyses.

With regards to relationship between advanced paternal age and risk of child behavioural problems, the few studies that exist present mixed results. In their general population birth cohort study, Saha *et al.* (2009a) found a linear relationship between paternal age and offspring externalizing behaviours, such that a 5-year increase in paternal age was related to a 12% increase in risk of child externalizing behaviours when children were 7 years old. It must be noted that participants were recruited for this study between 1959 and 1965; given the trend towards delaying parenthood has occurred within the last three decades, the study’s findings may not generalize to more contemporary cohorts. A study of Israeli young adults found a similar pattern of results, such that older fatherhood was related to a decreased level of social functioning in offspring. Compared to offspring born when fathers were aged 25–29 years, offspring of fathers aged ≥45 years at birth were found to be 1.7 times more likely to have impaired social functioning (Weiser *et al.*, 2008). This study benefitted from controlling for a number of covariates including coparent age, socioeconomic status, and birth order. However, since this study excludes female offspring and reports child outcomes for 16- and 17-year-olds, the findings have limited generalizability to families with children in early to middle childhood.

Conversely, Tearne *et al.* (2015) found no evidence of a paternal age effect on risk for problem behaviours in offspring over six phases of data collection, when children were aged between 2 and 17 years. Moreover, when child behavioural outcomes were analysed with paternal age as a categorical variable, paternal age of 35–39 years at birth was associated with a marginally significant decreased risk of behavioural problems in offspring. Similarly, Boivin *et al.* (2009) found no differences in child behavioural problems between young, middle and older aged fathers, with the parents of 4- to 11-year-olds in all age groups reporting equally warm and caring family environments. Jadva *et al.* (2022) measured child adjustment via the Strengths and Difficulties Questionnaire (Goodman, 1997) and found the vast majority of children at age 5 years to be functioning well, with no significant differences in children’s adjustment by parental age group.

Studies of advanced maternal age at birth indicate a broadly protective effect of older motherhood on the risk of childhood internalizing behaviours, such as depression and anxiety, although the evidence is still somewhat mixed. Tearne *et al.* (2015)’s prospective study found that increasing maternal age at birth was associated with decreases in childhood internalizing behaviours after controlling for coparent age, maternal risky behaviour during pregnancy, maternal education, maternal ethnicity, and total family income. Similarly, Trillingsgaard and Sommer (2018) found older motherhood to be related to fewer emotional difficulties in offspring at ages 7 and 11. The same association was observed in a sample of British 5-year-olds (Sutcliffe *et al.*, 2012). Conversely, two studies (Boivin *et al.*, 2009; Zondervan-Zwijnenburg *et al.*, 2020) have found no association between maternal age at birth and child internalizing behaviours, and one study (Saha *et al.*, 2009a) found a positive linear association between maternal age at birth and offspring internalizing behaviours, such that older motherhood was associated with increased risk of offspring internalizing behaviours when the children were aged 7. It was suggested that parenting styles of older mothers may contribute to greater anxious symptomatology in offspring (Saha *et al.*, 2009a); this explanation is partially supported by qualitative studies of mothers’ representations of their parenting as more anxious and overprotective (Chen and Landau, 2015), and by findings demonstrating an association between perceived parental overprotectiveness and offspring anxious symptomatology (Gere *et al.*, 2012; Fulton *et al.*, 2014). However, the findings of Saha *et al.*’s study are again limited in their generalizability due to the time elapsed since the data were collected. The few studies that focus on older fatherhood have found no evidence of a paternal age effect on child internalizing behaviours (Boivin *et al.*, 2009; Saha *et al.*, 2009a; Tearne *et al.*, 2015; Zondervan-Zwijnenburg *et al.*, 2020).

## Advanced parental age and child cognitive ability

Studies examining the associations between parental age and child cognitive ability are somewhat inconclusive. Whilst the literature generally agrees that older maternal age is associated with better child cognitive outcomes, some studies suggest the association is linear, whereas others suggest it is curvilinear, with worse child outcomes associated with the extremes of maternal age. In their large birth cohort study, Fergusson and Lynskey (1993) found a consistent linear association between increasing maternal age and superior offspring cognitive performance when children were aged 8 years. However, regression analyses revealed that controlling for a number of sociodemographic correlates, including maternal education, socioeconomic status and family size, accounted for the vast majority of the observed relationships. More recently, Duncan *et al.* (2018) analysed data from the US National Longitudinal Study of Youth and found a similar linear association between advanced maternal age and child cognitive outcomes, with each year the mother delays a first birth being associated with a 0.02–0.04 standard deviation increase in school achievement. As this dataset included mothers who were sisters, and thus children who were siblings and cousins, the analysis was able to control for a wider range of often unobserved, time-invariant maternal and family environment variables that may correlate with maternal age at first birth (e.g. grandparents’ education level, shared genetic factors). The linear association between advanced maternal age and child academic ability remained present after controlling for this wide set of covariates. Mediational analyses suggested that the primary pathway between advanced maternal age at first birth and beneficial child outcomes was the mothers’ increased number of years spent in education. This positive linear relationship has also been found amongst large, contemporary Dutch samples (Veldkamp *et al.*, 2021).

Conversely, Goisis *et al.* (2017) analysed data from three UK cohort studies spanning almost 50 years. Analyses of the earlier cohorts (the National Child Development Study, 1958, and the 1970 British Cohort Study) demonstrated an inverted U-shaped association between maternal age and child cognitive ability, with children of mothers aged 25–29 demonstrating the best outcomes at age 10–11 years. However, analysis of the most recent cohort (the Millennium Cohort Study, 2000–2002) revealed that advanced maternal age was associated with better child cognitive outcomes. The cross-cohort differences largely disappeared after controlling for child birth order and sociodemographic factors including maternal education level and paternal social class. The authors suggest that, in more modern cohorts, advanced maternal age is associated with socioeconomic advantages, which may relate to more positive child developmental outcomes. Similarly, Falster *et al.* (2018) utilized regression risk modelling to estimate children’s (n = 99 530) risk of developmental vulnerability across a number of socioemotional and cognitive domains, according to mothers’ age at birth. With regards to children’s language, cognitive and communication skills, increasing maternal age was associated with a lower risk of developmental vulnerability for children born to mothers ages from around 15–30 years. Increasing maternal age from around 35 to 45 years was associated with increasing vulnerability, although not to the same level of risk as children of very young mothers (<20 years). Contemporary findings therefore remain inconclusive, with some evidence of a linear, and some evidence of a curvilinear, relationship between maternal age at birth and child cognitive outcomes.

Again, very few studies have examined an association between advanced paternal age and child cognitive ability. In contrast to their findings regarding advanced maternal age, Saha *et al.* (2009b) found that advanced paternal age was associated with poorer performance of their children on a battery of neurocognitive measures. These findings are corroborated by a recent study examining associations between advanced paternal age and 51 5- to 8-year-olds’ reading ability (Xia *et al.*, 2021). The study found that advanced paternal age significantly predicted children’s reading difficulty, even after controlling for a broad range of demographic and known risk factors, including maternal age, general intelligence, child age and sex, familial reading risk, and child cognitive-linguistic abilities (Xia *et al.*, 2021). The study also controlled for environmental factors such as family socioeconomic status, parental education level, and the literary environment at home and found that older paternal age independently predicted children’s reading difficulty. The limited empirical evidence therefore suggests that older fatherhood may be associated with poorer child cognitive outcomes, although this finding requires replicating with larger, contemporary samples.

## Directions for future research

Several studies have found LGBTQIA+ identifying parents to be older than their heterosexual counterparts (Mazrekaj *et al.*, 2020; Carone* etal.,* 2017), potentially due to the lowered risk of unplanned pregnancy, and the often costly and time-consuming process of pursuing MAR or adoption. Despite this, the vast majority of studies of advanced parental age focus on cisgender heterosexual couples (e.g. Boivin *et al.*, 2009; Saha *et al.*, 2009a,b; Jadva *et al.*, 2022), or otherwise do not report parents’ sexual orientations and gender identities (e.g. Fergusson and Lynskey, 1993; Tearne *et al.*, 2015; Trillingsgaard and Sommer, 2018). To our knowledge, the only evidence on psychosocial outcomes in relation to parental age in LGBTQIA+ families comes from Mazrekaj *et al.* (2020), who utilized data from the Dutch municipal register to examine children’s academic ability amongst families headed by a combined sample of same-sex female (n = 2786) and same-sex male (n = 185) couples. The study found that children of same-sex couples performed significantly better on a standardized test of academic ability at age 12 than children of different-sex couples. It was further found that children of older same-sex couples (age >45 years) performed better than children of same-sex couples in younger age groups (≤25, 26–30, 31–35, 36–40, and 41–45 years). No studies have examined parental psychological wellbeing, the quality of the parent–child relationship or child adjustment in families headed by older LGBTQIA+ parents, although the literature consistently shows that family processes, rather than family structure, is most important for family functioning and child adjustment (Golombok, 2015).

Similarly, parents who have elected to have children without a partner, often referred to as solo parents or single parents by choice, tend to be older than first-time parents in the general population, with solo mothers typically starting their families in their late 30s–early 40s (Mannis, 1999; Jadva *et al*., 2009; Golombok *et al*., 2021), and solo fathers typically becoming parents in their early-to-mid-40s (Carone *et al*., 2017; Jones *et al*., 2022). Despite this, very little is known about the psychosocial functioning in families headed by solo parents, and nothing is known about this specifically in relation to advanced parental age. The few studies that do investigate parent and child psychosocial wellbeing in solo mother by choice families suggest that mothers and children are functioning well in families headed by cisgender heterosexual (Murray and Golombok, 2005a,b; Golombok *et al*., 2021) and lesbian solo mothers (MacCallum and Golombok, 2004). The limited literature on psychosocial functioning in solo father families similarly demonstrates broadly positive adjustment (Carone *et al.*, 2020; Jones *et al.*, 2022). However, recent evidence suggests an association between solo fathers’ increased perceived stigma about family type and poorer parent–child interaction quality (Carone *et al*., 2021), suggesting that, as with older heterosexual parents, perceived stigma may have the capacity to adversely affect solo fathers’ psychological wellbeing and parenting behaviours. Whether single parents perceive stigma about their older age in addition to stigma regarding family type, and whether such stigma is related to family psychosocial functioning, is worthy of further investigation.

## Conclusions

The literature regarding the psychosocial wellbeing of older parents during the early childhood period is limited, but points to good psychological functioning in both older mothers and fathers. However, potential risk factors for family functioning in older parent families have also been identified, including the perception and experience of stigma, lower levels of available social support, and concerns about mortality. The few studies that have directly examined parent–child relationship quality in older parent families suggest positive functioning. However, those examining the relationship between advanced parental age and child adjustment are inconclusive, with associations found between advanced parental age and child problem behaviours in some, but not all, cases. Similarly, the evidence is somewhat mixed for the association between advanced maternal age and child cognitive ability; advanced maternal age appears to be associated with good child cognitive outcomes, at least to a certain extent, with some evidence of a diminishing association beyond the age of around 35 years. The evidence is limited regarding advanced paternal age and child cognitive outcomes, but points to an association between advanced paternal age and poorer child cognitive ability. The very limited evidence suggests that children from families headed by older LGBTQIA+ parents function well in terms of academic ability. The literature will benefit from more studies aiming specifically to address the psychosocial outcomes associated with advanced parental age amongst single parents by choice and in LGBTQIA+ populations.

Widespread variation in methodological approaches make it difficult to determine the unique effects of older parental age on family functioning, over and above factors such as the experience of infertility and treatment type. It is therefore important to note that studies of older parents amongst the general population may not be generalizable to parents who have used MAR to have their child; studies that take account of the different paths to parenthood are required to better understand the experiences and outcomes of older parents. Moreover, not all studies account for the various protective factors from which older parents may benefit, such as greater psychological maturity, financial security, education level and career stability. With the trend in older parenthood continuing to rise, understanding the outcomes for those involved will be even more crucial. A major gap in our understanding so far is in how children feel about being raised by older parents; understanding their perspectives will be important in evaluating the outcomes of older parenting.

## Data Availability

No new data were generated or analysed in support of this review.
